# Obesogenic diets alter metabolism in mice

**DOI:** 10.1371/journal.pone.0190632

**Published:** 2018-01-11

**Authors:** Megan R. Showalter, Eric B. Nonnecke, A. L. Linderholm, Tomas Cajka, Michael R. Sa, Bo Lönnerdal, Nicholas J. Kenyon, Oliver Fiehn

**Affiliations:** 1 NIH West Coast Metabolomics Center, University of California Davis, Davis, CA, United States of America; 2 Department of Nutrition, University of California Davis, Davis, CA, United States of America; 3 Department of Internal Medicine, Division of Pulmonary and Critical Care, University of California Davis, Davis, CA, United States of America; 4 Biochemistry Department, Faculty of Science, King Abdulaziz University, Jeddah, Saudi Arabia; Georgetown University, UNITED STATES

## Abstract

Obesity and accompanying metabolic disease is negatively correlated with lung health yet the exact mechanisms by which obesity affects the lung are not well characterized. Since obesity is associated with lung diseases as chronic bronchitis and asthma, we designed a series of experiments to measure changes in lung metabolism in mice fed obesogenic diets. Mice were fed either control or high fat/sugar diet (45%kcal fat/17%kcal sucrose), or very high fat diet (60%kcal fat/7% sucrose) for 150 days. We performed untargeted metabolomics by GC-TOFMS and HILIC-QTOFMS and lipidomics by RPLC-QTOFMS to reveal global changes in lung metabolism resulting from obesity and diet composition. From a total of 447 detected metabolites, we found 91 metabolite and lipid species significantly altered in mouse lung tissues upon dietary treatments. Significantly altered metabolites included complex lipids, free fatty acids, energy metabolites, amino acids and adenosine and NAD pathway members. While some metabolites were altered in both obese groups compared to control, others were different between obesogenic diet groups. Furthermore, a comparison of changes between lung, kidney and liver tissues indicated few metabolic changes were shared across organs, suggesting the lung is an independent metabolic organ. These results indicate obesity and diet composition have direct mechanistic effects on composition of the lung metabolome, which may contribute to disease progression by lung-specific pathways.

## Introduction

Obesity and metabolic syndrome are comorbidities often associated with various lung diseases, particularly asthma. However, there are many important outstanding questions that derive from these observations. It is still unclear how alterations in food intake or body mass index affect lung metabolism, and how differences in lung metabolite composition impact the persistence or progression of lung diseases like asthma. This knowledge gap creates challenges for clinicians looking to offer simple dietary recommendation to patients with complex diseases.

Obesity is characterized by increased deposition of adipose tissue, insulin resistance and a global increase in production of pro-inflammatory mediators and altered metabolism [1]. Experimental mouse models of diet-induced obesity have demonstrated structural and immunological changes in the airways of the lung [1–3]. Despite advances in the understanding of global mechanisms by which obesity and associated metabolic dysregulation may alter lung function, specific metabolic effectors underlying changes in pulmonary function remain to be fully characterized. It is also not clear how diet and obesity impact the normal lung and how differences in high calorie diet derived metabolic products might differentially affect the lung. Investigations into the role of diet on basal lung metabolism are warranted to better understand the epidemiological link between increased adiposity and airway disease, and we set out to address this knowledge gap.

Mouse and rat models of diet induced obesity have been used previously in studies addressing the link between adiposity and asthma. High fat and high fructose diets increased airway hyper-responsiveness, reduced exhaled nitric oxide production and decreased arginine bioavailability [3]. Mice fed chow containing high fat or high fructose underwent significant structural changes as observed by electron microscopy, specifically for mitochondria in bronchial epithelia. Such findings support the idea that dietary differences might affect the pathophysiology of obesity induced “asthmatic” mice at the cellular level [4]. Some studies have addressed surfactant metabolism and observed functional differences in the lung including modified functional responses to infection, injury and allergen challenge, possibly through altered metabolism[5]. Metabolites from lipid oxidation and markers of dyslipidemia have been found to significantly impact the pattern of lung injury and abnormal repair resulting from influenza infection [6]. Long-chain acylcarnitines have also been shown to reduce lung function through inhibition of adsorption of pulmonary surfactants [7]. These findings highlight possible mechanisms by which metabolic changes induced by obesity could alter lung physiology.

While it is known that certain mouse strains (e.g., C57BL/6) accumulate marked adiposity and associated metabolic dysbiosis when assigned to a high fat diet [8], to date, little is known regarding the influence of prolonged high fat and/or high sugar feeding on basal lung metabolism. Utilizing established models of high calorie feeding, we aimed to characterize features of the lung metabolome in response to dietary perturbations in order to better understand the baseline for the possible relationship between metabolic disorders and lung disease in humans. We performed a comprehensive metabolomic investigation into the lungs of mice fed control (CTRL), high fat/sugar (HFS) or very high fat (VHF) chows. This study was designed to characterize metabolic features of diet assignment that could contribute to altered responses to disease in the lung. Inclusion of two obesogenic diet models with varied compositions of fat (i.e., lard) and sugar (i.e., sucrose) enabled a multi-layered approach to metabolic dysregulation associated with obesogenic diets. We hypothesized the metabolic derangement associated with obesity would have metabolic impacts on the lung independent of the liver and kidney, classic regulators of metabolism. We surmised that dietary composition could contribute to altered responses of the lung to the environment.

To test our hypothesis, we used untargeted metabolomic and lipidomic analyses. Metabolism receives input from all levels of regulation including genotype, protein biochemistry, small molecule levels and environmental exposures. Comprehensive metabolic assessments can therefore be thought of as a measurement of the true phenotype of the cell [9]. Metabolites also serve functions beyond their canonical roles in catabolism and anabolism, including signaling and regulatory functions of non-metabolic processes. We define these non-canonical metabolites as epimetabolites [10]. It is essential to consider changes in the metabolome, including epimetabolites, as contributors to diet induced lung changes.

## Results

C57BL/6 mice fed high fat/sugar (HFS) or very high fat (VHF) chow diets (Table 1) gained more weight across the study period than control (CTRL) fed animals (Table 2). Feeding HFS and VHF chow resulted in similar weight gain and body fat accumulation despite variations in fat and sugar content, and caloric contributions. To yield a comprehensive view of metabolic changes associated with diet assignment, we performed untargeted metabolomic and lipidomic analysis on lung, liver and kidney tissues. We utilized gas chromatography–time-of-flight mass spectrometry (GC-TOFMS), hydrophilic interaction liquid chromatography–quadrupole/time-of-flight mass spectrometry (HILIC-QTOFMS) and reversed-phase liquid chromatography–quadrupole/time-of-flight mass spectrometry (RPLC-QTOFMS) to increase coverage of metabolites spanning diverse chemical classes. Overall, we annotated or identified [11] 447 distinct metabolites, in addition to detecting many unknown metabolic signals. GC-TOFMS data was processed using BinBase [12] and LC-QTOFMS data using MS-DIAL software [13]. Data were normalized to the sum of all identified metabolites, log transformed, Pareto scaled and subjected to multivariate and univariate statistical analysis.

**Table 1:** Diet composition. Male C57BL/6N mice, ages 6–7 weeks, were fed control (CTRL), high-fat sugar (HFS), or a very high fat (VHF) diet (Research Diets, NJ) for approximately 150 days, with n = 8 for each diet. Diets were matched for vitamin and mineral content, and varied only in energy composition.

	CTRL (D12450K)	HFS (D12451)	VHF (D12492)
**kcal/g**	3.85	4.73	5.24
**Fat (% kcal)**	10.0	45.0	60.0
**Sucrose (% kcal)**	0.0	17.0	7.0

**Table 2:** Body weight and food intake across the study period. At necropsy, various tissues were collected from terminally anesthetized animals, including lung, liver, kidneys and adipose depots. Total adipose depot weight is comprised of subcutaneous adipose (i.e., inguinal and shoulder adipose fat pads), and intra-abdominal depots, including epididymal, mesenteric, and perirenal fat pads. Chow mass was measured daily and *n* = 8 for each diet, numbers reflect averages and standard deviation for each group.

	CTRL	HFS	VHF
**Weight gained (g)**	14.5 ± 2.1	21.7 ± 2.6	23.5 ± 1.7
**Total body adipose (mg)**	531.4 ± 101.62	907.5 ± 79.48	1013.5 ± 125.3
**Food intake (g/d)**	2.9 ± 0.1	2.6 ± 0.2	2.4 ± 0.2

**Fig 1:**
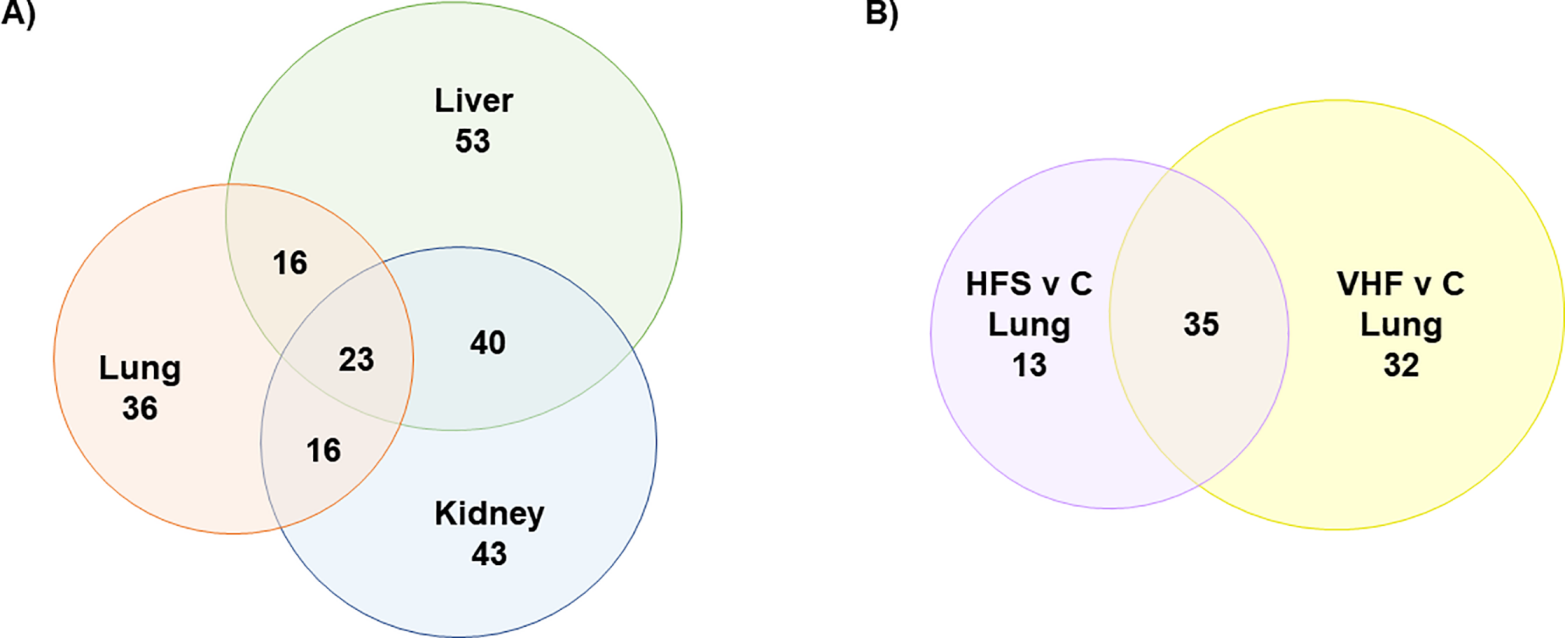
**Distribution and number of structurally identified or annotated metabolites found to be significantly altered by dietary intervention with raw *p*<0.05 in all dietary comparisons in all tissues measured (A) and in the lung by obesogenic diet compared to control (B).** A: In total 91, 122 and 132 metabolites were significantly altered in the lung, kidney and liver respectively. B: In the lung, 80 metabolites were significantly altered in HFS and VHF compared to control diet, with 35 compounds significantly altered between both obese groups and control group. Significance determined using student’s *t*-test with *p-*value <0.05 as significant cut off and *n* = 8.

We first tested if either of the two dietary interventions resulted in significant metabolic changes in each of the three organs, and if the same compounds would be found to be regulated in two or more organs. Interestingly, each organ showed quite different metabolic regulation in response to the dietary interventions. The Venn diagrams in Fig 1A shows most diet-induced metabolic differences found were tissue specific. We focused here on structurally annotated metabolites only. Overall, 228 of 447 structurally annotated metabolites were found to be altered by dietary differences in comparison to control chow and between obese groups in any of the organs. A complete list of significantly changed metabolites with *p*-values and fold-changes are listed in S1 File. However, only 23 compounds (5%) were found to be differentially regulated in all three organs. Instead, 30% of the regulated metabolites were specific for only one organ and not found significantly altered by dietary intervention in other organs. When focusing on lung metabolism only, 36 compounds (40%), were found distinctly different in the lung after dietary challenges but not found to be altered in other tissues (Fig 1A). Indicating both global and organ specific changes occurred due to diet assignment. Comparison between each of the two obese animal groups and the control group in the lung are shown in Fig 1B. In the lung 35 (44%) of metabolites were significantly altered between both obese groups and the control group, highlighting that both obesity and diet specific changes occurred. Diet specific changes were indicated by metabolites only significantly altered in one diet which were 13 (16%) in HFS compared to control and 32 (40%) in VHF compared to control.

We then tested if the observed metabolic differences were sufficient to yield clear overall metabolic phenotypes. Multivariate analysis projects overall variation in metabolite abundances to visualize global metabolic differences between treatment groups. Unsupervised principal component analysis (PCA) showed no overt metabolic phenotypes in lung metabolism induced by either HFS or VHF diets (S3 and S4 Figs), meaning that overall natural metabolic variance in lung metabolism was higher than overall differences induced by dietary interventions. As expected, metabolic phenotypes in kidney and liver tissues were overtly different by dietary intervention when analyzed by total variance-based PCA (data not shown). In contrast, supervised regression models (orthogonal projections to latent structures discriminant analysis, OPLS-DA) clearly detected differences in metabolic phenotypes in lungs, kidneys and livers that were associated with dietary changes, using any of the three metabolomics platforms (S1 and S2 Figs), as expected by the number of specific metabolic changes found in univariate analysis.

**Fig 2:**
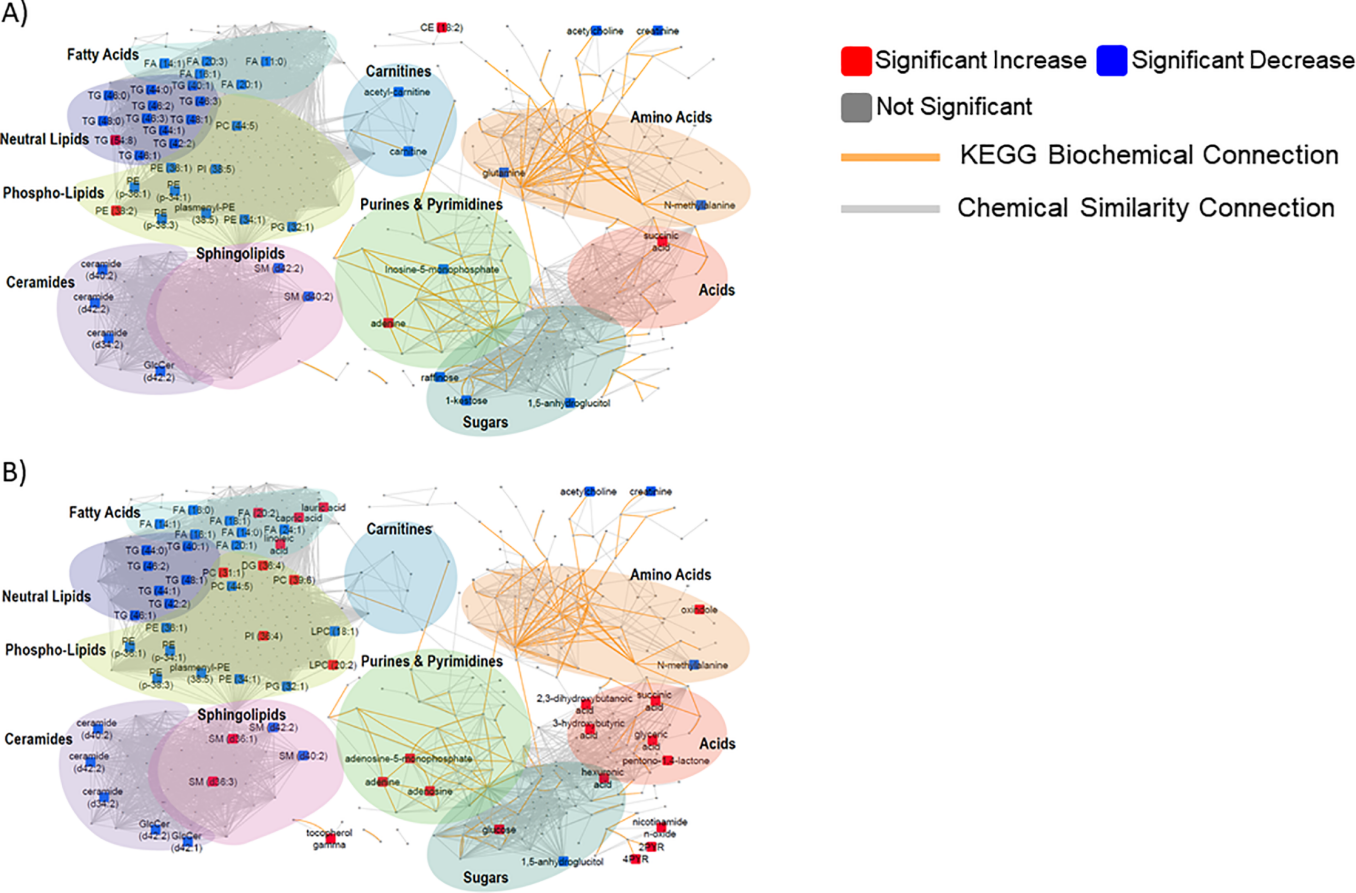
Metabolic network maps of lung tissue metabolomic and lipidomic results. **Metabolic modules indicated by colored regions using chemical similarity.** A) Metabolic differences in lung metabolism of mice fed High Fat Sugar (HFS) diet compared to samples from mice fed with control chow. B) Metabolic differences in lung metabolism of mice fed Very High Fat (VHF) diet compared to samples from mice fed with control chow. Significant metabolites shown in color with direction of indicated by color. Significance determined using student’s *t*-test with *p-*value <0.05 considered significant and *n* = 8.

These overall differences justified using detailed univariate investigations to build hypotheses regarding metabolic pathways that were specifically altered in lung metabolism during dietary regimen changes. In lung tissues, we found 21 primary metabolites that were significantly different due to HFS- or VHF-induced obesity, in addition to 18 biogenic amines and 52 lipids at raw significance values of *p*<0.05. In order to highlight biochemical overrepresentations, we mapped all metabolites and metabolic differences onto biochemical networks constructed using chemical and biochemical similarities in MetaMappR [14] (Fig 2). These networks use the KEGG reactant pairs database as foundation for biochemical similarity and Tanimoto substructure composition matrices for chemical similarity mapping. Hence, clusters of compounds visible in these networks represent larger biochemical modules, such as “amino acids”, “sugars”, “phospholipids” or “neutral lipids”. Such modules have the advantage to truly represent all identified metabolites in a data set, while direct pathway mapping (e.g., to KEGG Atlas maps [15]) leaves out many identified metabolites, especially lipids. Overall, the networks showed that obesity-induced differences in lung metabolism were not randomly distributed across metabolism, but focused on specific metabolic modules. Surprisingly, both diets showed a significant decrease in a range of lipids rather than an increase as one would expect from high-fat diets. Especially neutral lipids were deposited at lower amounts in lung tissues, along with decreases in unsaturated free fatty acids, and decreases in unsaturated phospholipids and unsaturated ceramides (Fig 2).

**Fig 3:**
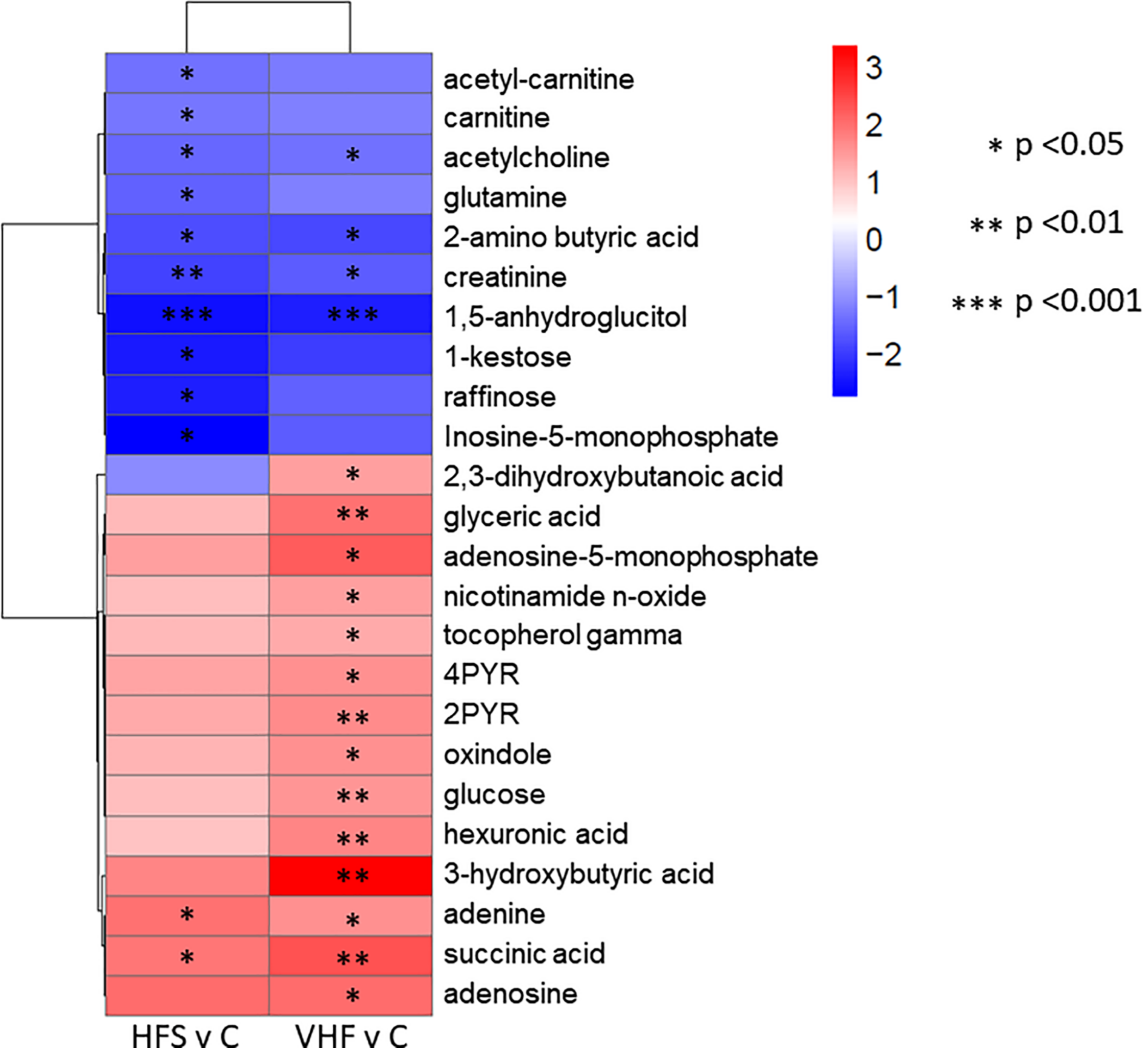
Heat-map of significant polar and primary metabolites with fold change and significance levels in the lung by diet compared to control. Metabolites were measured using GC-TOFMS and HILIC-QTOFMS and raw *p*-values from student’s *t*-test <0.05 were used for significance levels. *P*-values for each metabolite are indicated by asterisks. Averages for each compound by group are shown to indicate fold change magnitude and direction with *n* = 8.

**Fig 4:**
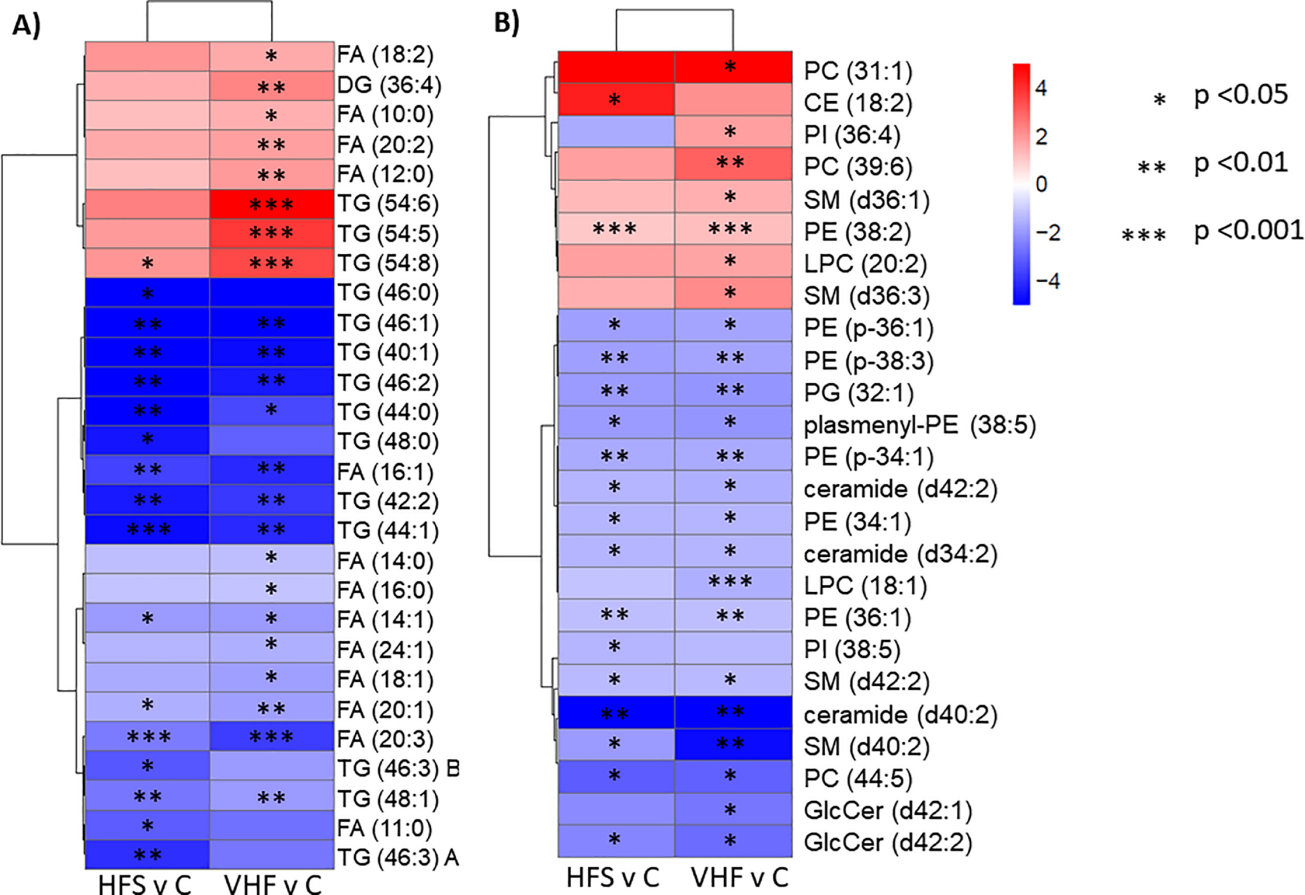
Heat-map of significant glycerophospholipids, fatty acids and sphingolipids with fold change and significance levels in the lung by diet compared to control. Lipid species were measured using RPLC-QTOFMS and raw *p*-values from student’s *t*-test <0.05 were used to determine significance levels. *P*-value of each lipid species are indicated by asterisks. Mean direction of change is indicated by color and intensity, with red representing increased values compared to control, and blue decreased values compared to control. Averages for each compound by group are shown with *n* = 8.

In comparison, other metabolic modules were less affected or remained unchanged. The VHF diet increased metabolic levels of lung ketone bodies in addition to succinate and several hydroxy acids (Fig 2B). In comparison to lungs from mice fed control diet or HFS diet, the VHF diet also resulted in increases in medium chain free fatty acids, linoleic acid and dihomo-γ-linolenic acid while still being decreased in contents of other free fatty acids including eicosaenoic acid or oleic acid (Fig 2B). In contrast, the HFS diet showed decreases in carnitine and acetyl-carnitine as well as trisaccharides (Fig 2A) that were absent after VHF diet modulation of metabolism. Additionally, the VHF diet showed a clearer induction of adenosine-pathway metabolism compared to the observed HFS metabolic phenotype (Fig 2). Figs 3 and 4 highlight lung metabolites with raw *p*-value <0.05 from student’s *t*-test, showing fold change and direction and hierarchical clustering of compounds.

**Fig 5:**
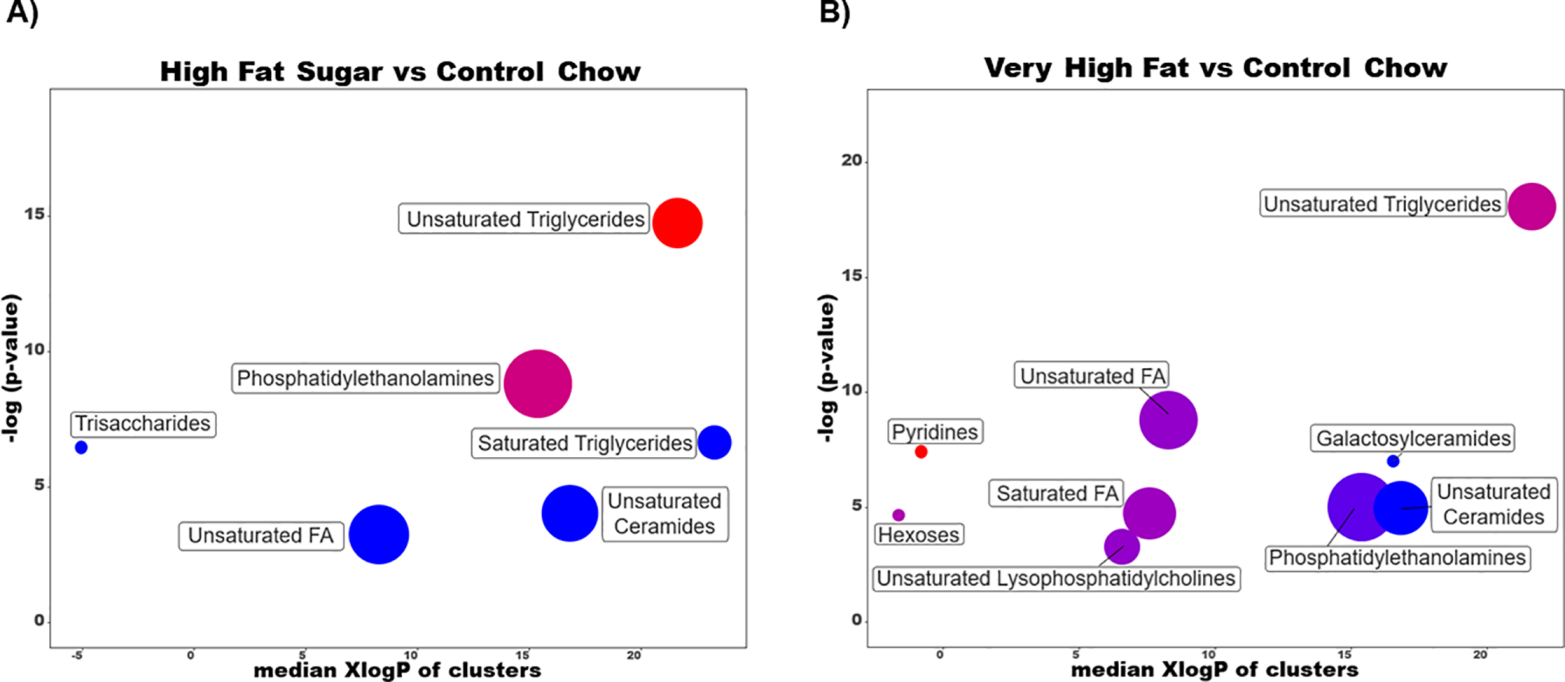
Chemical similarity enrichment analysis of annotated metabolites in both obesogenic diets compared to control chow. Statistical enrichment analysis utilized chemical similarity and ontology mapping to generate metabolite clusters. The *y*-axis shows most significantly altered clusters on top, *x*-axis shows XlogP values of metabolite clusters. Cluster colors give the proportion of increased or decreased compounds (red = increased, blue = decreased) in each cluster. Chemical enrichment statistics is calculated by Kolmogorov–Smirnov test. Only enrichment clusters are shown that are significantly different at *p* < 0.05. Plots and calculations were done using ChemRICH.

We performed chemical enrichment analysis to provide chemical classes significantly altered in each diet compared to control in Fig 5 using ChemRICH [16]. ChemRICH provides enrichment analysis based upon chemical structure and not defined pathways which can be inherently flawed and does not rely upon background databases for statistical calculations [16]. In HFS diet compared to control trisaccharides, unsaturated fatty acids, unsaturated ceramides and saturated triglycerides were significantly decreased while phosphatidylethanolamines were significantly altered with some species increased, others decreased. Unsaturated triglycerides were significantly increased. In VHF diet compared to control unsaturated ceramides were also decreased. Phosphatidylethanolamines were also significantly altered with increased and decreased trends, similar to HFS compared to control. Unsaturated triglycerides, unsaturated and saturated fatty acids and hexoses also showed increased and decreased significantly altered compounds. Pyridines showed significant increases in VHF compared to control. Significance was calculated using Kolmogorov–Smirnov test and only clusters with *p-*values <0.05 were shown in Fig 5.

## Discussion

An increasing number of epidemiological studies and well-designed mouse model studies suggest a complex interaction between lung diseases, particularly asthma, and obesity [8]. We utilized two obesogenic diets to induce obesity with the aim to study the independent effects of diet composition and obesity on the lung metabolome. We found metabolites significantly altered in the lung common to, and distinct between the obese groups. To our surprise, the most changes observed in the lung were not seen in liver and kidney tissues. Specific changes in the lung may contribute to the development or persistence of lung diseases such as asthma or chronic obstructive pulmonary disease (COPD), or to systemic conditions like metabolic syndrome. Interestingly, we demonstrate two mouse groups fed two different obesogenic diets both showed similar weight gains but were quite different in the resulting changes in lung metabolism. Overall more changes in lung metabolism were observed in the mouse group fed very high fat (VHF) diets compared to high fat/sugar diet (HFS). Nevertheless, many of the differences that showed raw significant values in the VHF diet at least also showed a trend in the same direction, albeit of non-significant *p*-values, in the HFS. Below, we highlight several groups of metabolites that were significantly different in the lung between diets.

### Differences in energy metabolites

The two experimental obese groups shared loss of blood sugar control and changes in global lipidomic profiles. Apart from these high-level similarities, there were numerous differences in glucose metabolism, the tricarboxylic acid (TCA) cycle and fatty acid metabolism, the main energy sources of the cell. 1,5-Anhydroglucitol was significantly decreased in both HFS-lung and VHF-lungs. 1,5-Anhydroglucitol is a validated glycemic control marker and reflective of glycemic control and glycemic excretion [17]. β-Hydroxybutyrate (βOHB), a ketone body, is produced from acetoacetate and used to transport energy to tissues with high energy demands during fasting or during prolonged exercise. βOHB was significantly increased in the VHF compared control lungs but this effect was not seen in the HFS diet group. It was unexpected to find βOHB not significantly increased in the HFS compared to control group as these mice also experienced decreased blood sugar control and similar adiposity. Recently, βOHB has been shown to act in non-canonical roles including histone deacetylase (HDAC) inhibition [18] and as a signaling molecule [19]. HDAC inhibition plays a critical role in inflammation of COPD [20, 21]. These epimetabolite functions of βOHB have been tied to regulation of inflammatory intermediates, autophagy, insulin signaling and lipolysis [19] and point to numerous ways the differential regulation of this metabolite could affect lung health, particularly in individuals with high fat diets.

Both creatinine and glutamine, related to energy metabolism, were found to be differentially regulated in the lung compartment compared to liver and kidneys. Glutamine, an amino acid that can feed into the TCA cycle for energy metabolism, was found to be significantly decreased in the HFS-lung compared to both control and VHF groups. The lung has been previously reported to be key in maintaining systemic glutamine homeostasis [22]. It is possible that HFS diet significantly disrupted glutamine homeostasis in a matter different than the VHF diet in the lung. This could point to a specific mechanism by which diet composition, and not obesity alone, differentially affect lung, and systemic metabolism. It appears the changes to glutamine in the HFS-lung are compartment specific, and the consequences or causes of reduced glutamine remain unclear.

Creatinine, the spontaneous breakdown product of cellular creatine, was significantly decreased in both HFS (*p*-value 0.007) and VHF (*p*-value 0.04) lungs and not significantly changed in liver or kidney tissues. Creatine is produced in a two-step enzymatic reaction by arginine:glycine amidinotransferase (AGAT) and guanidinoacetate *N*-methyltransferase (GAMT), primarily in liver and kidney. In the lung AGAT but not GAMT activity has been reported and creatine transporters showed little to no expression levels [23]. Clinically, creatinine is used as a marker for renal function and protein metabolism. The reduction in creatinine might also point to a generic decrease in the amount of muscle mass that could contribute to asthmatic symptoms including declining in lung function.

Glucose was significantly increased in the VHF-lung compared to both HFS and control. The significant increase in glucose but not glucose 6-phosphate, indicates a reduction in commitment of glucose to metabolism through glycolysis. Increased free glucose can then enter the aldose reductase pathway (polyol pathway) leading to decreased lung health by two possible mechanisms [24]. First, the polyol pathway reaction consumes NADPH and NAD^+^, preventing use for important pathways such as glutathione and nitric oxide production. Second polyol pathway intermediates are used in the non-enzymatic glycosylation of protein and collagen residues called Advanced Glycation End products (AGEs). The glycosylation of these amino acid residues leads to disruption of protein function and activation of receptors for AGEs (RAGEs) which activate pro-inflammatory cytokines. The polyol pathway is known to become activated in patients with uncontrolled blood sugar and has been implicated as a main driver in the formation of diabetes-induced oxidative stress [25]. Glucose was not found to be significantly changed in either kidney or liver tissues. Fructose and fructose phosphates showed varying non-significant trends in the lung.

### Differences in fatty acid metabolism

Metabolites involved in fatty acid β-oxidation were also differentially altered between the two obese groups, including free fatty acids (FFA) as shown in Fig 4A. Four FFA, specifically FAs (14:1), (16:1), (20:1) and (20:3) were significantly decreased in both VHF and HFS-lungs compared to control. Palmitoleic acid (FA (16:1)), is an omega-7 monounsaturated fatty acid and has been shown to improve insulin sensitivity and suppress inflammation[26]. Eicosatrienoic acid (FA (20:3)), also known as dihomo-γ -linolenic acid (DGLA), is an omega-6 fatty acid. DGLA can be converted into prostaglandin E1 (PGE1) which exerts a vasodilatory effect and can inhibit production of arachidonic acid derived eicosanoids. These free fatty acids, among others have been explored as treatments for allergic bronchial asthma [27]. The significant decrease in palmitoleic acid and eicosatrienoic acid may in part provide a possible mechanism by which obesity could contribute to lung disease progression due to increases in inflammation.

Additional metabolites involved in fatty acid metabolism showed varying trends between VHF and HFS-lungs. Carnitine and acetyl carnitine (C2) were significantly decreased in the HFS compared to control lung and butyryl carnitine (C4) was significantly increased in VHF compared to HFS-lung. The significant decreases in carnitine and short chain acylcarnitines in the HFS-lung are unexpected as there have been numerous reports of modest to large increases of carnitine and acylcarnitines in plasma of patients with T2DM [28, 29]. It is thought that these species accumulate due to reduced capacity for β-oxidation, or incomplete oxidation which can interfere with insulin signaling [30]. However, plasma concentrations of acylcarnitines are not predictive of tissue concentrations [30] and little is known regarding levels in the lung.

Acylcarnitines are formed by the esterification of a free fatty acid (FFA) to carnitine where fatty acid chain length provides information regarding the parent lipid source. Odd chain fatty acids of three or five carbons can be synthesized *de novo* from amino acids, whereas both amino acid and fatty acid catabolism can produce butyryl carnitine (C4). Acetyl carnitine (C2) is generated during carbohydrate metabolism and as an end-product of β-oxidation [28, 29]. As elevated carnitine could indicate incomplete β-oxidation of fatty acids, decreased carnitine and acetyl carnitine in the HFS-lung could indicate increased fatty acid β-oxidation. Alternatively, the reduction in acylcarnitine levels could indicate a decrease of mitochondrial CoA levels, as the mitochondrial carnitine/acylcarnitine carrier protein functions bidirectionally and acylcarnitines are a qualitative indicator of type and concentration of tissue CoA pools [29]. While long chain acylcarnitines were not significantly altered in the lung, they were significantly increased in both VHF and HFS-livers compared to control. The disruption of FFA β-oxidation is important to lung heath as accumulation of acylcarnitines, specifically long chain acylcarnitines have been shown to disrupt pulmonary surfactants *in vitro* [7].

The lung lipidome was significantly altered in both obese lungs compared to the control group with the majority of lipids significantly altered in the same direction in both obese groups compared to control as shown in Fig 4. We looked to pulmonary surfactant related lipids including phosphatidylcholine (PC), phosphatidylglycerol (PG) and phosphatidylethanolamine (PE) species[31, 32] for specific changes that could affect lung function. We found non-significant changes in major surfactant PC lipid species. Instead, a range of minor surfactant phospholipids were found differentially regulated. PG 32:1 (16:0_16:1), PE species PE (34:1), PE (36:1), PE (p-34:1), PE (p-36:1), PE (p-38:3), and PE (p-38:5) were significantly decreased in the HFS and VHF-lungs while PE (38:2) was significantly increased in both obese groups compared to control. In total, 25 PE species were measured, with 8 significantly altered in the lung in both obese groups compared to the control group. From these results it is clear obesity, and not specific diet composition, is contributing to dysregulation of these species in the lung. Sphingomyelin and ceramide species showed varying significant changes in the two obese groups compared to control, and have also been tied to changes in lung function[33]. Triacylglycerols (TGs) were found to be significantly different between the obese and control groups with the majority of significantly altered species decreased in the obese lung compared to control. The impact of decreased TGs in the lung remains unclear. Overviews of lipid class chemical enrichment analysis are shown in Fig 5. Overall, the lung lipidome appears to be largely influenced by obesity independent of diet composition, and the direct role these changes may play in lung health remains largely unexplored.

### Differences in antioxidant and nucleotide metabolism

Metabolites related to nucleotide metabolism were found to be significantly altered in the VHF and HFS-lungs compared to controls. In the VHF-lung purines adenine, adenosine and adenosine-5-monophosphate (AMP) were all significantly increased. Adenosine signaling has been implicated in chronic lung diseases. Signaling occurs through release of adenosine and activation of adenosine receptors which then activate cascades that can influence tissue remodeling and inflammation. The response to adenosine releases appear to be different between acute and chronic lung injuries and the resulting purinergic remodeling has been proposed to affect progression of disease. It is known that both AMP and adenosine are released upon tissue damage, which are sources of excess adenosine for signaling activation [34]. Allantoic acid is the terminal step in purine nucleotide metabolism in most mammals and is known to be increased with the addition of adenosine. Allantoic acid has also been reported as a marker for oxidative stress *in vivo*. It is disputed if allantoic acid, reported in humans in multiple studies, is an endogenous metabolite: however, transcripts for allantoicases, which produce allantoic acid, have been reported in both mice and humans [35]. Allantoic acid was significantly increased in VHF-lung compared to HFS-lung and could indicate increased purine metabolism or increased oxidative stress in the VHF-lung. The increases in these metabolites highlight an already altered state of this pathway due to diet alone without lung injury or disease.

In VHF-lungs compared to control three metabolites related to nicotinamide metabolism were found to be significantly increased. Nicotinamide and NAD are important compounds for cellular function and NAD serves as a cofactor for numerous reactions. However, from the metabolites found to be altered, clear dysfunction is occurring in this pathway. Nicotinamide *N*-oxide is a precursor for NAD production while *N*^1^-methyl-2-pyridone-5-carboxamide (2PYR) and *N*^1^-methyl-4-pyridone-3-carboxamide (4PYR) are the metabolites of 1-methylnicotinamide (1MNA). The production of 1MNA by nicotinamide *N*-methyltransferase (NNMT) is proposed to contribute to diet induced obesity through regulation of NAD and ultimately Sirt1 signaling. NNMT knockout was found to protect against diet induced obesity proposed to be through the regulation of NAD and *S*-adenosylmethionine (SAM) levels [36]. 1MNA and NNMT also regulate cellular methylation status, as 1MNA is used as a methyl sink by cells to control SAM levels [37]. It appears that in the VHF-lung both upregulating the production and sink of NAD molecules is occurring. From chemical enrichment analysis, this compound class, pyridines, were significantly increased in VHF compared to control lungs (Fig 5). These compounds, including 1MNA were significantly increased also in the liver and kidney, showing potentially multi-organ dysregulation of this pathway. The impact of dysregulation of this pathway both globally, and on the lung, remains unclear.

## Conclusion

In summary, we found significant global metabolic alterations in the lung, distinct from those found in the liver and kidney. These results establish the lung as organ affected by diet and the metabolic derangements of obesity. Pathways of significantly altered metabolites emerged in the lung, some with commonalities between obese groups and others showing varying changes. We report alterations in metabolites of the TCA cycle, antioxidant and nucleotide pathways as described in detail above. The lung lipidome showed significant changes in neutral lipids, fatty acids, phospholipids and sphingolipids. These differences suggest possible mechanisms by which metabolic changes induced by obesity could alter lung physiology. The significant changes in obese lungs highlight the altered state of the un-challenged obese lung and could provide mechanisms by which diet influences disease progression. Many metabolites significantly altered in this study have not been reported previously in the lung and it is therefore clear more research on the metabolic role of the lung is needed to better understand the impact of these changes.

## Methods

### Animal and diet information

Male C57BL/6N mice, ages 6–7 weeks, were fed control (CTRL), high-fat sugar (HFS), or a very high fat (VHF) diet (Table 1, Research Diets, NJ) for approximately 150 days. Body weight and food intake was recorded across the study period. At necropsy, various tissues were collected from terminally anesthetized animals, including lung, liver, kidneys and adipose depots. Mice were euthanized under deep anesthesia with a cocktail of ketamine and xylazine (100/10 mg/kg). Total adipose depot weight is comprised of subcutaneous adipose (i.e., inguinal and shoulder adipose fat pads), and intra-abdominal depots, including epididymal, mesenteric, and perirenal fat pads and shown in Table 2. All procedures involving live animals were approved by the Institutional Animal Care and Use Committee at the University of California, Davis. Mice were housed in a temperature and humidity controlled room on a 12 h light:dark cycle and provided free access to food and water.

### Sample preparation for GC-TOFMS analysis

Six milligrams of frozen lung, liver and kidney tissue was ground using a GenoGrinder 2010 (SPEX SamplePrep) for 2 min at 1350 rpm. Homogenized tissue was then extracted with 1 ml of a degassed acetonitrile:isopropanol:water (3:3:2, v/v/v) mixture (Fisher) at −20°C and centrifuged at 14,000 rcf. All supernatants were removed and evaporated to dryness using a CentrVap. To remove membrane lipids and triglycerides, dried samples were resuspended with 0.5 mL of an acetonitrile:water (1:1, v/v) mixture, decanted and evaporated to dryness using a CentrVap. Samples were derivatized with 10 μL of methoxyamine hydrochloride in pyridine (40 mg/mL) by shaking at 30°C for 90 min followed by trimethylsilylation with 90 μL of *N*-methyl-*N*-(trimethylsilyl) trifluoroacetamide (MSTFA, Sigma-Aldrich) by shaking at 37°C for 30 min containing C8–C30 fatty acid methyl esters (FAMEs) as internal standards. Derivatized samples were subsequently submitted for analysis by GC-TOFMS (0.5 μl injection).

### Chromatographic and mass spectrometric conditions for GC-TOFMS analysis

Primary metabolite data was collected using a Leco Pegasus IV time-of-flight (TOF) MS (Leco Corporation) coupled to an Agilent 6890 GC (Agilent Technologies) equipped with a 30 m long 0.25 mm id Rtx-5Sil MS column (0.25 μm film thickness) and a Gerstel MPS2 automatic liner exchange system (Gerstel GMBH & Co. KG). The chromatographic gradient used a constant flow of 1 ml/min with following gradient: 50°C (1 min), 20°C/min to 330°C, hold 5 min. Mass spectrometry data was collected using 1525 V detector voltage at *m*/*z* 85–500 with 17 spectra/s, electron ionization at −70 eV and an ion source temperature of 250°C. QC injections, blanks and pooled human plasma were used for quality assurance throughout the run. Data was processed by ChromaTOF for deconvolution, peak picking, and BinBase [12] for metabolite identifications.

### Sample preparation for LC-QTOFMS analysis

Six milligrams of frozen lung, liver and kidney tissue was ground using a GenoGrinder 2010 (SPEX SamplePrep) for 2 min at 1350 rpm. Homogenized tissue was then extracted with 225 μl of methanol at −20°C containing an internal standard mixture of PE(17:0/17:0), PG(17:0/17:0), PC(17:0/0:0), C17 sphingosine, ceramide (d18:1/17:0), SM (d18:0/17:0), palmitic acid-*d*_3_, PC (12:0/13:0), cholesterol-*d*_7_, TG (17:0/17:1/17:0)-*d*_5_, DG (12:0/12:0/0:0), DG (18:1/2:0/0:0), MG (17:0/0:0/0:0), PE (17:1/0:0), LPC (17:0), LPE (17:1), and 750 μL of MTBE (methyl tertiary butyl ether) (Sigma Aldrich) at −20°C containing the internal standard cholesteryl ester 22:1. Samples were shaken for 6 min at 4°C with an Orbital Mixing Chilling/Heating Plate (Torrey Pines Scientific Instruments). Then 188 μl of LC-MS grade water (Fisher) was added. Samples were vortexed, centrifuged and the upper (non-polar) and bottom (polar) layers were collected (350 μL and 125 μL, respectively) and evaporated to dryness.

The non-polar layer was re-suspended in a methanol:toluene (9:1, v/v) mixture containing 50 ng/ml CUDA ((12-[[(cyclohexylamino)carbonyl]amino]-dodecanoic acid) (Cayman Chemical) while the polar layer was resuspended in an acetonitrile:water (4:1, v/v) mixture with 5 μg/ml Val-Try-Val (Sigma). Samples were then vortexed, sonicated for 5 min and centrifuged and prepared for lipidomic or polar metabolite analysis. Method blanks and pooled human plasma (BioreclamationIVT) were included as quality control samples.

### Chromatographic and mass spectrometric conditions for lipidomic RPLC-QTOF analysis

For analysis of the non-polar phase, re-suspended samples were injected at 1 μL and 5 μL for ESI positive and negative modes respectively, onto a Waters Acquity UPLC CSH C18 (100 mm length × 2.1 mm id; 1.7 μm particle size) with an additional Waters Acquity VanGuard CSH C18 pre-column (5 mm × 2.1 mm id; 1.7 μm particle size) maintained at 65°C was coupled to an Agilent 1290 Infinity UHPLC (Agilent Technologies). To improve lipid coverage, different mobile phase modifiers were used for positive and negative mode analysis [38]. For positive mode 10 mM ammonium formate and 0.1% formic acid were used and 10 mM ammonium acetate (Sigma–Aldrich) was used for negative mode. Both positive and negative modes used the same mobile phase composition of (A) 60:40 v/v acetonitrile:water (LC-MS grade) and (B) 90:10 v/v isopropanol:acetonitrile. The gradient started at 0 min with 15% (B), 0–2 min 30% (B), 2–2.5 min 48% (B), 2.5–11 min 82% (B), 11–11.5 min 99% (B), 11.5–12 min 99% (B), 12–12.1 min 15% (B), and 12.1–15 min 15% (B). A flow rate of 0.6 mL/min was used. For data acquisition an Agilent 6550 QTOF with a jet stream electrospray source with the following parameters was used: mass range, *m*/*z* 50–1700; capillary voltage, ±3 kV; nozzle voltage, ±1 kV; gas temperature, 200°C; drying gas (nitrogen), 14 L/min; nebulizer gas (nitrogen), 35 psi; sheath gas temperature, 350°C; sheath gas flow (nitrogen), 11 L/min; acquisition rate, 2 spectra/s. For lipid identification, MS/MS spectra were collected at a collision energy of 20 eV with an acquisition rate MS^1^ of 10 spectra/s (100 ms) and an acquisition rate for MS/MS of 13 spectra/s (77 ms) with 4 precursor ions per cycle. Mass accuracy was maintained by constant reference ion infusion (purine and HP-0921 in an acetonitrile:water mixture).

### Chromatographic and mass spectrometric conditions for polar metabolite HILIC-QTOFMS analysis

Hydrophilic interaction liquid chromatography (HILIC) method was used for analysis of the polar phase. Five microliters of re-suspended sample was injected onto a Waters Acquity UPLC BEH Amide column (150 mm length × 2.1 mm id; 1.7 μm particle size) with an additional Waters Acquity VanGuard BEH Amide pre-column (5 mm × 2.1 mm id; 1.7 μm particle size) maintained at 45°C coupled to an Agilent 1290 Infinity UHPLC. The mobile phases were prepared with 10 mM ammonium formate and 0.125% formic acid (Sigma–Aldrich) in either 100% LC-MS grade water for mobile phase (A) or 95:5 v/v acetonitrile:water for mobile phase (B). Gradient elution was performed from 100% (B) at 0–2 min to 70% (B) at 7.7 min, 40% (B) at 9.5 min, 30% (B) at 10.25 min, 100% (B) at 12.75 min, isocratic until 16.75 min with a column flow of 0.4 mL/min. Spectra were collected using a TripleTOF 5600+ (SCIEX, Framingham, MA, USA) using data dependent mode for MS/MS spectra acquisition. Data was collected in ESI(+) mode with a mass range of *m*/*z* 50–1700. Other parameters were curtain gas: 35, ion source gas 1 and 2: 60, temperature: 350°C, ion spray voltage floating: +4.5 kV, declustering potential: 80 V. DDA MSMS parameters were MS^1^ accumulation time 100 ms, MS^2^ accumulation time 50 ms, dependent product ion scan number 8, intensity threshold 1000, active precursor exclusion after 2 spectra for 5 s, collision energy 20 eV with 15 eV collision energy spread. Mass accuracy was maintained through calibration performed automatically after every 11 injections using APCI positive calibration solution delivered using a calibration delivery system.

### LC-MS data processing using MS-DIAL and statistics

Both lipidomic and HILIC data processing was performed using MS-DIAL [13] for deconvolution, peak picking, alignment, and identification. For both data sets, in house *m/z* and retention time libraries were used in addition to MS/MS spectra databases in msp format [39]. Features were reported when present in at least 50% of samples in each group. Statistical analysis was done by first normalizing data using the sum of the knowns, or mTIC normalization, to scale each sample. Peak heights were then submitted using R to DeviumWeb. The data was normalized further by log transformation and Pareto scaling. ANOVA analysis was performed with FDR correction and post hoc testing. PCA analysis was used for multivariate statistics and visualization. For network mapping, KEGG reactant pairs and Tanimoto similarity calculations were done using MetaMapR [14]. Networks were then created using Cytoscape [40]. Chemical enrichment calculations were done using ChemRICH [16].

## Supporting information

S1 File Compound IDs, student’s *t*-test results and fold change for all annotated compounds. (XLSX)

S1 Fig Orthogonal partial least square discriminant analysis (OPLSDA) of HILIC and RPLC-QTOFMS metabolite data of lung, liver and kidney tissues. Validation statistics by100 rounds of Monte Carlo permutation testing and for A: Lung Q2 = 0.0495, RSMEP = 0.0198, B: Liver Q2 = 0.0099, RSMEP = 0.0198 and C: Kidney Q2 = 0.0099, RSMEP = 0.0198.
(TIF)

S2 Fig Orthogonal partial least square discriminant analysis (OPLSDA) of GC-TOFMS metabolite data of lung, liver and kidney tissues. Validation statistics by100 rounds of Monte Carlo permutation testing and for A: Lung Q2 = 0.2673, RSMEP = 0.0396, B: Liver Q2 = 0.0693, RSMEP = 0.1089 and C: Kidney Q2 = 0.2970, RSMEP = 0.1386.
(TIF)

S3 Fig Principle component analysis (PCA) of HILIC and RPLC-QTOFMS metabolite data of lung, liver and kidney tissues. (TIF)

S4 Fig Principle component analysis (PCA) of GC-TOFMS metabolite data of lung, liver and kidney tissues. (TIF)
