# Supplementary figures and images for: Obesogenic diets alter metabolism in mice
Source: PLoS One. 2018 Jan 11;13(1):e0190632. doi: 10.1371/journal.pone.0190632 (PMC5764261; doi:10.1371/journal.pone.0190632)

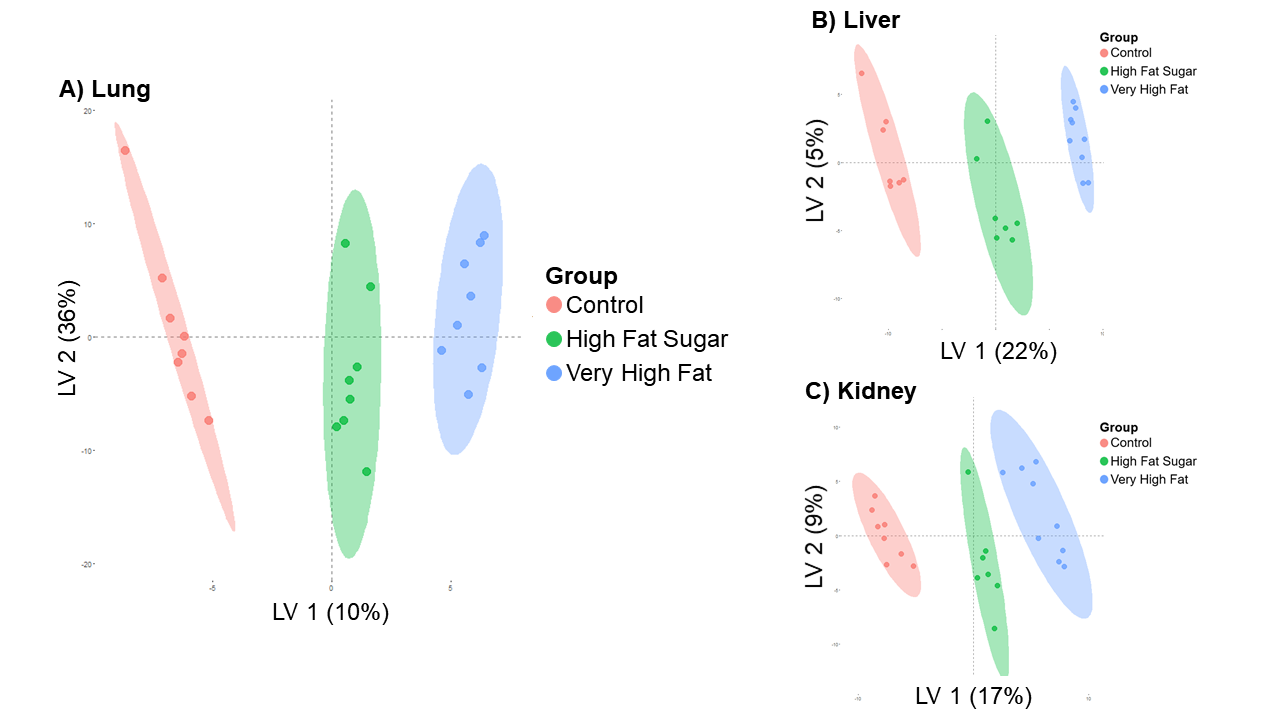

Supplement: S1 Fig — Validation statistics by100 rounds of Monte Carlo permutation testing and for A: Lung Q2 = 0.0495, RSMEP = 0.0198, B: Liver Q2 = 0.0099, RSMEP = 0.0198 and C: Kidney Q2 = 0.0099, RSMEP = 0.0198. (TIF) [file pone.0190632.s002.tif]

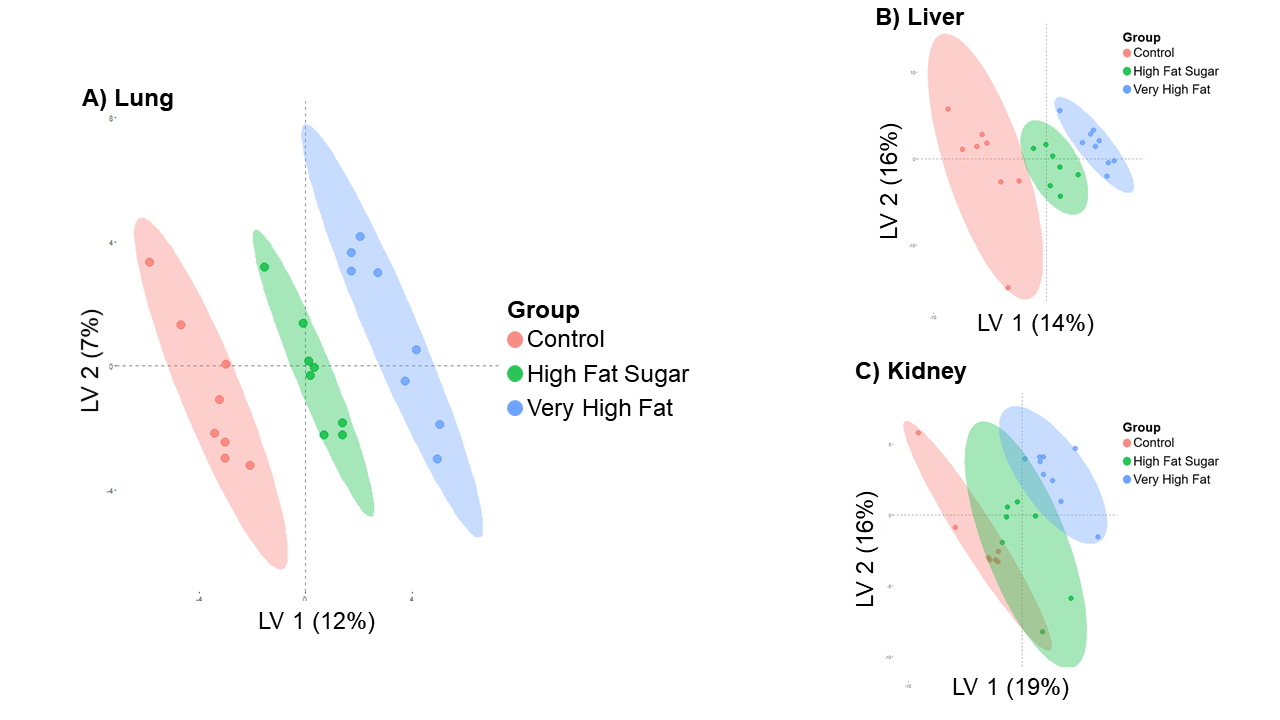

Supplement: S2 Fig — Validation statistics by100 rounds of Monte Carlo permutation testing and for A: Lung Q2 = 0.2673, RSMEP = 0.0396, B: Liver Q2 = 0.0693, RSMEP = 0.1089 and C: Kidney Q2 = 0.2970, RSMEP = 0.1386. (TIF) [file pone.0190632.s003.tif]

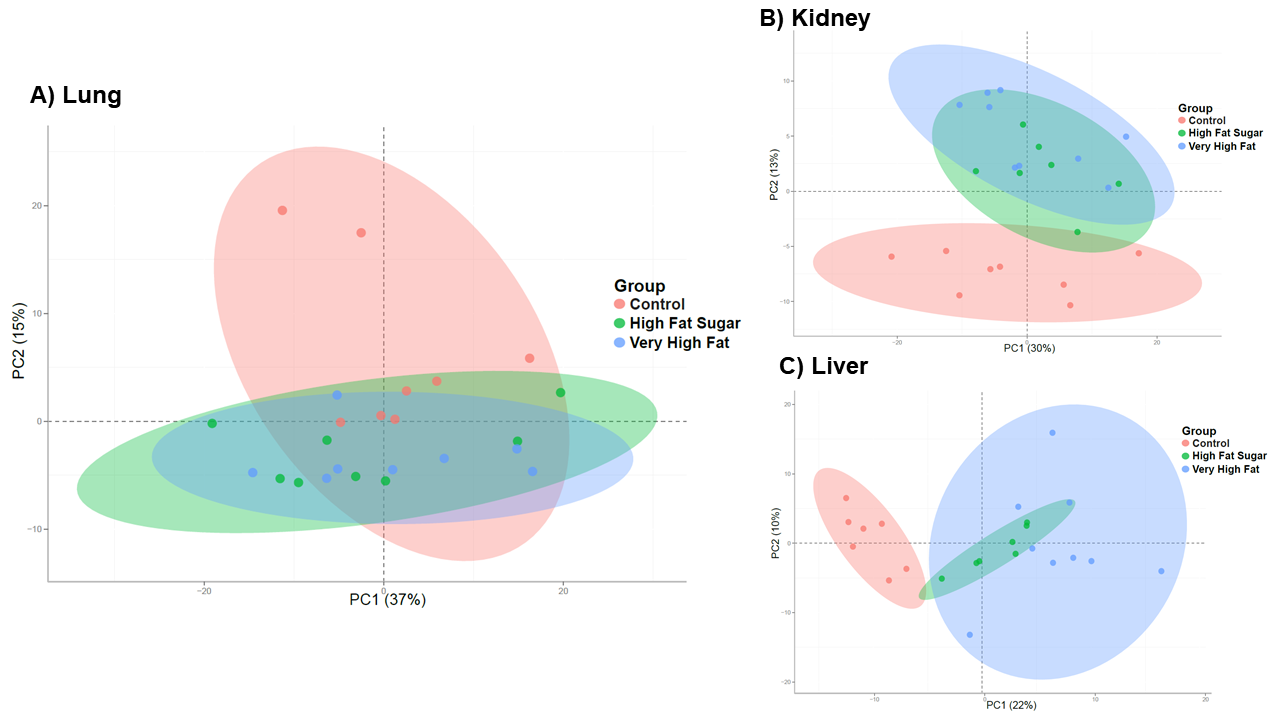

Supplement: S3 Fig — (TIF) [file pone.0190632.s004.tif]

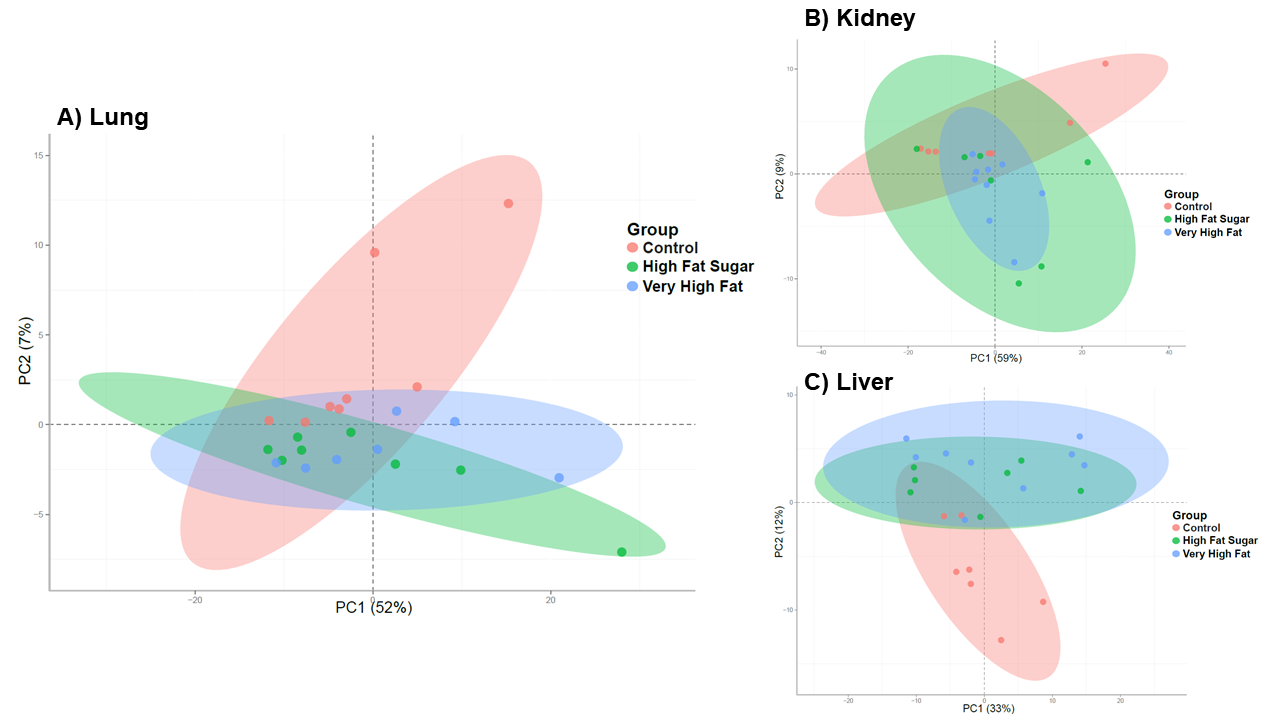

Supplement: S4 Fig — (TIF) [file pone.0190632.s005.tif]
